# Optimization of Adventitious Root Suspension Culture in *Valeriana fauriei* and GC-MS-Based Metabolomics of Responses to Methyl Jasmonate

**DOI:** 10.3390/plants15091382

**Published:** 2026-04-30

**Authors:** Yihan Qian, Ping Song, Jing Wen, Meiyang Li

**Affiliations:** College of Agriculture, Yanbian University, Yanji 133002, China; 2022010604@ybu.edu.cn (Y.Q.); 2021010604@ybu.edu.cn (P.S.); 18943733795@163.com (J.W.)

**Keywords:** medicinal plant, in vitro propagation, uniform design, elicitation, differential metabolites

## Abstract

To overcome the depletion of wild resources and limited seed propagation of *Valeriana fauriei*, an adventitious root suspension culture system was established and optimized, and methyl jasmonate (MeJA)-elicited metabolic responses were then evaluated using gas chromatography-mass spectrometry (GC-MS)-based untargeted metabolomics. Culture conditions were optimized using a single-factor medium screening experiment combined with a uniform design for four culture-condition factors. The results showed that the optimized culture system increased the 28 d proliferation coefficient of *Valeriana fauriei* adventitious roots to 27.07. A total of 181 significant differential metabolites were identified and classified into four clusters according to their changing trends. The first cluster increased and then decreased, reaching the maximum value at 80 mg·L^−1^ MeJA. The second cluster decreased and then increased, reaching the minimum value at 80 mg·L^−1^ MeJA. The third cluster increased, then decreased, and then increased again, reaching the maximum value at 40 mg·L^−1^ MeJA and the minimum value at 160 mg·L^−1^ MeJA. The fourth cluster increased continuously with increasing MeJA concentration. Subsequently, KEGG pathway enrichment analysis was performed for the metabolite sets of the four clusters. Combined analysis indicated that 80 mg·L^−1^ MeJA was the treatment concentration that most strongly affected the metabolic biosynthesis of *V. fauriei* adventitious roots. Under this treatment, pathways related to membrane transport, amino acid metabolism, translation, nucleotide metabolism, and the biosynthesis of other secondary metabolites were the most significantly enriched.

## 1. Introduction

*Valeriana fauriei* Briq. is a perennial herb of the genus *Valeriana* in the family Valerianaceae. It is a medicinal *Valeriana* species native to Northeast China, Japan, and Korea [1,2]. There are 436 confirmed species in the genus *Valeriana* worldwide. Most of them are distributed in regions with mild and humid climates across Europe, northern Asia, South America, and the United States. About 30 species, including varieties, occur in China, mainly in the southwest and northeast regions [3,4].

Plants of the genus *Valeriana* have been used medicinally for more than 2000 years, and the roots are commonly used as medicine. *Valeriana officinalis* L. was first used as a sedative and was later found to be useful for epilepsy, splenic disorders, gynecological diseases, nervous tension, headache, and palpitations [5]. Although *V. fauriei* has only been recorded in the Japanese Pharmacopoeia as a substitute for *V. officinalis* [6], previous studies showed that its binding activity to the GABAA receptor is markedly higher than that of *V. officinalis*, suggesting that it may have a stronger sedative effect [7]. In addition, *V. fauriei* has been reported to have antidepressant, analgesic, sleep-improving, prenatal stress-relieving, and neurodevelopmental disorder-preventive effects [8,9,10,11,12,13]. Because of overharvesting and environmental damage, wild valerian resources have sharply declined. In natural conditions, valerian seeds also show low germination rates, which limits large-scale seed propagation and also makes seedling breeding difficult [14,15]. Another major factor limiting large-scale development of *V. fauriei* cultivation is that *Valeriana* species are easily affected by cultivation area and climatic conditions such as temperature, which leads to unstable production of key bioactive constituents [15,16]. Because the medicinal materials of *Valeriana* species are derived mainly from underground organs, establishing a stable root-based in vitro culture system is important for the medicinal development of this genus [3]. At present, studies on *V. fauriei* remain limited. However, studies on other *Valeriana* species have shown the great potential of in vitro adventitious root culture to shorten the growth cycle and increase the accumulation of medicinal constituents [17]. Li et al. [18] and Gehlot et al. [17] studied *V. officinalis* and *Valeriana jatamansi*, respectively, and found that liquid suspension culture can markedly shorten the growth cycle of root materials in these two species while providing relatively stable accumulation of active constituents.

Methyl jasmonate (MeJA) is a plant lipid-derived jasmonate that is widely present in nature and acts as an endogenous growth regulator in many plants [19,20]. As an important regulator involved in many physiological and biochemical processes, MeJA plays key roles in plant growth and development, including the induction of stress and defense responses and the promotion of secondary metabolite synthesis [21,22]. Previous studies found that, compared with elicitors such as chitosan, salicylic acid, and yeast extract, MeJA has a more significant effect on the accumulation of active constituents in *Valeriana* plants [23]. At present, metabolomics has been used to analyze the response patterns of plant tissues induced by MeJA [24,25]. Gas chromatography–mass spectrometry (GC–MS) is suitable for the analysis of volatile constituents and small-molecule metabolites after derivatization, and has been widely applied in studies on the active constituents of *Valeriana* species [26,27]. For example, Pokharel et al. [27] used GC-MS to compare root components of *Valeriana jatamansi* Jones collected at different altitudes.

In recent years, sleep disorders, anxiety, and depression have increasingly affected human health in modern society [28]. Although benzodiazepines and related sedative drugs act quickly, long-term use may lead to adverse effects such as residual daytime sedation, cognitive impairment, increased risk of falls, tolerance, and dependence. In comparison, valerian root extracts may have broader application prospects. The sustained availability of *Valeriana* root materials with stable yield, consistent origin, and high levels of active constituents is a prerequisite for the development and industrialization of this botanical medicine [28,29].

Therefore, this study used repeatedly subcultured root segments of *V. fauriei* as materials. First, a suspension culture system for efficient adventitious root culture was developed by single-factor experiments and a uniform design to improve biomass growth efficiency. Different concentrations of MeJA were then applied under the optimized culture system, and GC-MS-based untargeted metabolomics was used to analyze the metabolic responses of *V. fauriei* adventitious roots, with the aim of providing technical support and a theoretical basis for improving metabolite accumulation in these roots.

## 2. Results and Analysis

### 2.1. Optimization of Adventitious Root Proliferation Culture of Valeriana fauriei Briq.

#### 2.1.1. Effects of Different Media on Adventitious Root Proliferation

After 28 d of culture, the proliferation coefficients of adventitious roots differed significantly among the four media, with the order WPM > N6 > White > MS; WPM showed the highest proliferation coefficient, 25.01, whereas MS showed the lowest, 0.88 (Table 1).

**Figure 1:**
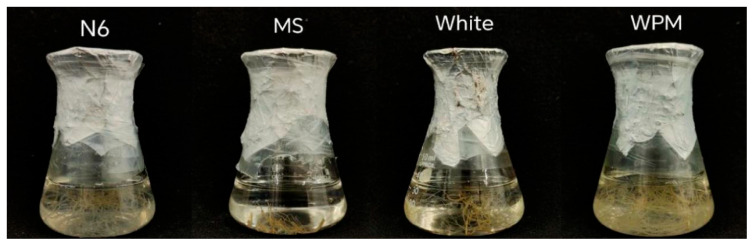
Growth status of adventitious roots of *Valeriana fauriei* cultured in different media. WPM, Woody Plant Medium; MS, Murashige and Skoog medium; White, White medium; N6, Chu’s N6 medium.

In addition, the growth status of adventitious roots in WPM was clearly better than that in the other three media. Therefore, WPM was selected as the basal medium for the subsequent suspension culture experiments (Figure 1).

#### 2.1.2. Results of the Uniform Design for Adventitious Root Proliferation

According to the method described in Section 4.4.1, the variables *x*_1_, *x*_2_, *x*_3_, and *x*_4_ were modeled, and the regression equation was obtained as Equation (1).

Sucrose concentration (g·L^−1^), total nitrogen concentration (mmol·L^−1^), total phosphorus concentration (mmol·L^−1^), and salt solution concentration (the original formulation was set as *x*) were defined as *x*_1_, *x*_2_, *x*_3_, and *x*_4_, respectively. After data preprocessing, multiple linear regression analysis was performed to establish the regression equation and evaluate the applicability of the model. Modeling of *x*_1_, *x*_2_, *x*_3_, and *x*_4_ by multiple linear regression generated Equation (1).

y = 10.5 + 0.25 *x*_1_ +0.15 *x*_2_ − 2.5 *x*_3_ + 1.8 *x*_4_ (1)

The coefficient of determination of the regression model was R^2^ = 0.85, indicating that the model explained 85% of the variation in y and showed a good fit. The F-test gave *p* < 0.05, indicating that the regression equation was significant as a whole. The *t*-test results are shown in Table 2. Variables *x*_1_, *x*_2_, and *x*_4_ had significant effects on y (*p* < 0.05), whereas the effect of *x*_3_ was not significant (*p* = 0.085), but it was still retained in the model.

The model was further tested for multicollinearity, normality, independence, and homogeneity of variance. The VIF values of *x*_1_ to *x*_4_ were 2.1, 1.9, 3.2, and 2.5, respectively, all below 5, indicating no serious multicollinearity. The Shapiro–Wilk test gave *p* > 0.05, indicating that the residuals met the normality assumption. The Durbin–Watson statistic was DW = 1.85, which was close to 2 and indicated no autocorrelation among the residuals. The Bartlett test gave *p* > 0.05, indicating homogeneity of variance. These results suggested that the regression model was reliable and suitable for further analysis.

Equation (2) was optimized by gradient descent. The final optimal parameters were *x*_1_ = 42, *x*_2_ = 45, *x*_3_ = 1.5, and *x*_4_ = 1.8. Substituting these values into the regression equation gave a theoretical optimum of y* = 27.24. For easier operation, the validation experiment was carried out under the following conditions: sucrose 42 g·L^−1^, total nitrogen 44.8 mmol·L^−1^, total phosphorus 1.5 mmol·L^−1^, and salt solution concentration 1.8×. Using the inoculation method described in Section 4.2.1 and the culture conditions above, six replicate experiments were performed. The observed proliferation coefficient was 27.07 ± 2.71, which differed from the theoretical value by only 0.17, further supporting the reliability of the model.

### 2.2. Metabolomic Analysis

#### 2.2.1. Sample Comparison Analysis

**Figure 2:**
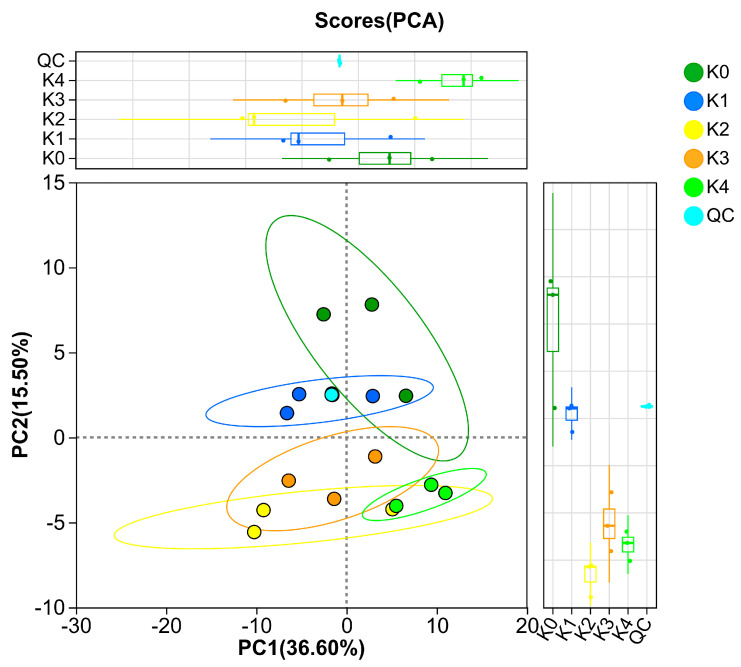
Principal component analysis (PCA) score plot with marginal boxplots for control (K0), MeJA-treated samples (K1–K4), and QC samples based on all detected metabolites. Distances between points reflect the similarity among samples. The 95% confidence ellipses indicate the distribution range of each group, and marginal boxplots show the distribution of sample scores along the PC1 and PC2 axes.

After preprocessing of the data from the five sample groups, 331 compounds were identified in total. Among them, 112 metabolites were annotated in KEGG, 123 were annotated in HMDB, and 84 were assigned to KEGG pathways. Figure 2 shows the principal component analysis (PCA) based on all detected metabolites and presents the overall separation trend of the samples. The cumulative explained variance of PC1 and PC2 was R^2^X(cum) = 0.52, indicating that the PCA model had good explanatory power. As shown in Figure 2, the QC samples clustered at one point, and no obvious outlier was observed in any treatment group, suggesting that the analytical system was stable and that the error was small. In addition, the boxplots of PC1 and PC2 showed that the separation among groups on both axes changed with MeJA concentration, indicating that changes in MeJA concentration did affect the metabolism of adventitious roots of *V. fauriei* (Figure 2).

#### 2.2.2. Analysis of Differential Metabolites

**Figure 3:**
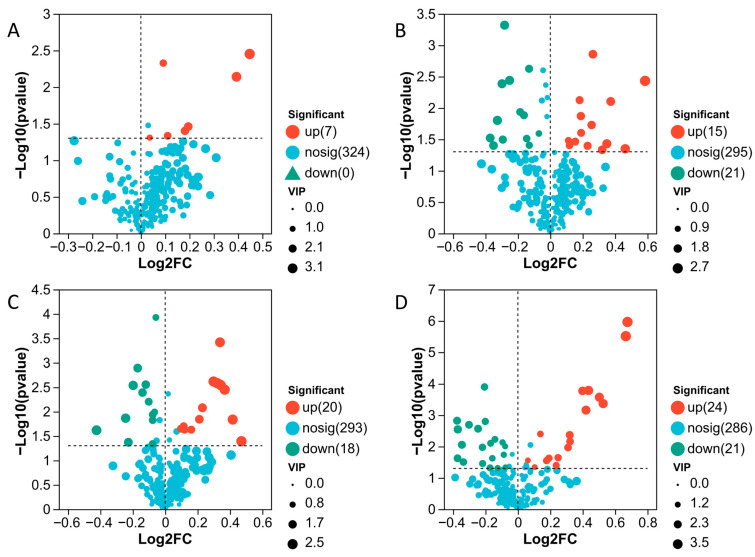
Volcano plots of differential metabolites in different MeJA treatment groups compared with K0. (**A**) K1 vs. K0; (**B**) K2 vs. K0; (**C**) K3 vs. K0; (**D**) K4 vs. K0. The x-axis represents the fold change of metabolites between two groups (log_2_FC), and the y-axis represents the statistical significance of differences in metabolite abundance (−log_10_(*p*-value)). Both axes are log-transformed. Each point corresponds to an individual metabolite, and the point size indicates the VIP value. Red points represent significantly upregulated metabolites, green points represent significantly downregulated metabolites, and blue points indicate metabolites with no significant differences. After mapping all metabolites, points on the left indicate downregulated metabolites, whereas points on the right indicate upregulated metabolites. Metabolites located further toward the horizontal extremes and higher on the vertical axis exhibit greater differential significance.

Differential metabolites were identified using the thresholds of |log2FC| ≥ 1 and *p* < 0.05. Relative to K0, the numbers of up-regulated metabolites in K1 (40 mg·L^−1^), K2 (80 mg·L^−1^), K3 (160 mg·L^−1^), and K4 (320 mg·L^−1^) were 8, 15, 24, and 29, respectively, whereas the corresponding numbers of down-regulated metabolites were 0, 26, 20, and 24. As MeJA concentration increased, the number of up-regulated metabolites rose gradually. By contrast, once the concentration exceeded 80 mg·L^−1^, the number of down-regulated metabolites remained comparatively high (Figure 3).

**Figure 4:**
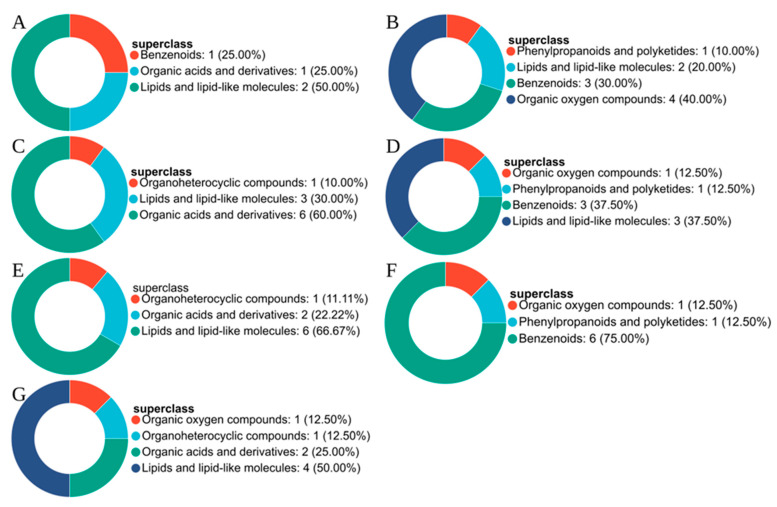
HMDB compound classification of differential metabolites in different MeJA treatment groups compared with K0. (**A**) upregulated compounds in K1 vs. K0; (**B**) downregulated compounds in K2 vs. K0; (**C**) upregulated compounds in K2 vs. K0; (**D**) downregulated compounds in K3 vs. K0; (**E**) upregulated compounds in K3 vs. K0; (**F**) downregulated compounds in K4 vs. K0; (**G**) upregulated compounds in K4 vs. K0.

The metabolites shown in Figure 3 were subsequently assigned to HMDB categories. In the comparison between K1 and K0, no down-regulated metabolites were detected. The up-regulated metabolites were represented mainly by lipids and lipid-like molecules (50%), while the remainder consisted primarily of benzenoids and organic acids and their derivatives (Figure 4A). In K2 relative to K0, organic oxygen compounds and benzenoids constituted the major down-regulated categories, accounting for 40% and 30%, respectively, whereas the remaining down-regulated metabolites belonged to lipids and lipid-like molecules and phenylpropanoids and polyketides (Figure 4B). Among the up-regulated metabolites in this comparison, organic acids and their derivatives formed the largest category, followed by lipids and lipid-like molecules and organic heterocyclic compounds (Figure 4C).

For K3 vs. K0, the down-regulated metabolites were distributed mainly between benzenoids and lipids and lipid-like molecules, each contributing 37.5%, with the remaining compounds classified as phenylpropanoids and polyketides and organic oxygen compounds (Figure 4D). In contrast, the up-regulated metabolites were dominated by lipids and lipid-like molecules (66.67%), while the rest were assigned to organic acids and their derivatives and organic heterocyclic compounds (Figure 4E). In K4 vs. K0, benzenoids represented the largest proportion of down-regulated metabolites (75%), and the remaining compounds were mainly phenylpropanoids and polyketides and organic oxygen compounds (Figure 4F). Among the up-regulated metabolites, lipids and lipid-like molecules remained the predominant class (50%), followed by organic acids and their derivatives (25%), with the remaining compounds falling into the categories of organic oxygen compounds and organic heterocyclic compounds (Figure 4G).

**Figure 5:**
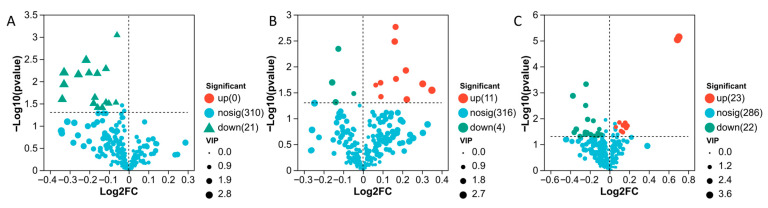
Volcano plots of differential metabolites in adventitious roots of *Valeriana fauriei* among adjacent MeJA treatment groups. (**A**) K2 vs. K1; (**B**) K3 vs. K2; (**C**) K4 vs. K3. The x-axis represents the fold change of metabolites between two groups (log_2_FC), and the y-axis represents the statistical significance of differences in metabolite abundance (−log_10_(*p*-value)). Both axes are log-transformed. Each point corresponds to an individual metabolite, and the point size indicates the VIP value. Red points represent significantly upregulated metabolites, green points represent significantly downregulated metabolites, and blue points indicate metabolites with no significant differences. After mapping all metabolites, points on the left indicate downregulated metabolites, whereas points on the right indicate upregulated metabolites. Metabolites located further toward the horizontal extremes and higher on the vertical axis exhibit greater differential significance.

To further examine the trends in metabolites with changing MeJA concentration, differences between adjacent groups were compared on the basis of the comparisons between each treatment group and the control. K2 versus K1 was dominated by down-regulation, whereas K3 versus K2 and K4 versus K3 both showed coexistence of up-regulation and down-regulation (Figure 5).

**Figure 6:**
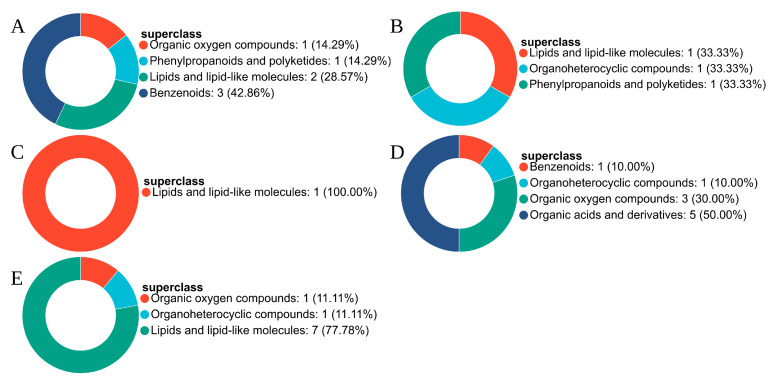
HMDB-based compound classification of differential metabolites among adjacent MeJA treatment groups. (**A**) downregulated compounds in K2 vs. K1; (**B**) downregulated compounds in K3 vs. K2; (**C**) upregulated compounds in K3 vs. K2; (**D**) downregulated compounds in K4 vs. K3; (**E**) upregulated compounds in K4 vs. K3. The percentages are displayed to two decimal places; therefore, they may not sum to exactly 100% due to rounding.

Compared with K1, K2 showed significant decreases in benzenoids, phenylpropanoids and polyketides, and lipids and lipid-like molecules (Figure 6A). Compared with K2, K3 also showed decreases in lipids and lipid-like molecules and in phenylpropanoids and polyketides, while some lipids and lipid-like molecules were up-regulated at the same time (Figure 6B,C). Compared with K3, the down-regulated compounds in K4 contained higher proportions of organic acids and their derivatives and organic oxygen compounds, whereas the remaining compounds were benzenoids and organic heterocyclic compounds. At the same time, lipids and lipid-like molecules and organic oxygen compounds were significantly up-regulated (Figure 6D,E).

Overall, different concentrations of methyl jasmonate mainly affected the accumulation of benzenoid compounds, lipids and lipid-like molecules, and organic acids and their derivatives in adventitious roots of *V. fauriei*. Treatment with 40 mg·L^−1^ MeJA promoted the accumulation of multiple metabolites without showing an inhibitory effect, whereas MeJA at 80 mg·L^−1^ and above produced a more complex regulatory effect on metabolite accumulation and requires further analysis.

#### 2.2.3. Cluster Analysis of Major Differential Metabolites

**Figure 7:**
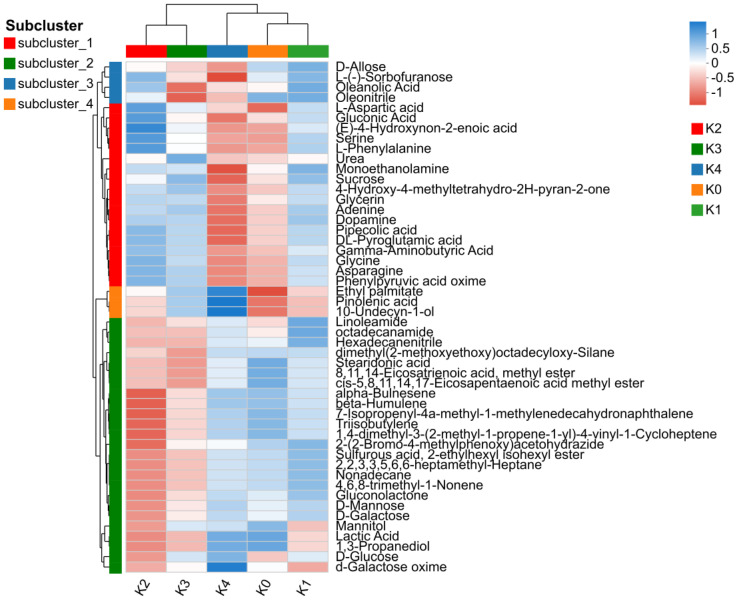
Cluster Heatmap of the Top 50 Differential Metabolites.

Based on the screening and classification above, a metabolite set containing 181 differential metabolites was established, and the top 50 metabolites with higher significance were used for cluster analysis. The dendrogram at the top grouped K2 and K3 together, indicating that the metabolic patterns of adventitious roots of *V. fauriei* were similar under medium MeJA concentrations. K4 was grouped with K0 and K1, suggesting that the metabolic pattern of the high-concentration treatment shared some similarity with those of the control and low-concentration groups. The dendrogram on the left divided these 50 metabolites into four clusters, which were named subcluster 1–4 (Figure 7).

**Figure 8:**
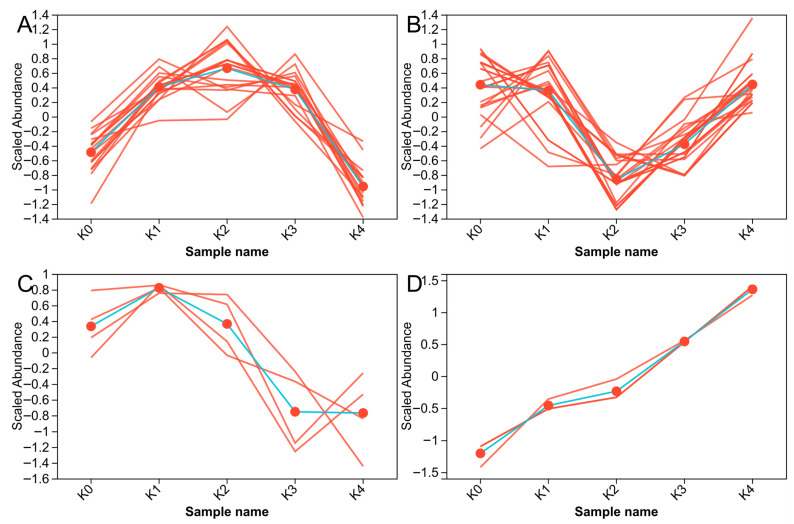
Trend plots of the four metabolite subclusters. (**A**) Subcluster 1; (**B**) Subcluster 2; (**C**) Subcluster 3; (**D**) Subcluster 4. To investigate the relative variation trends of metabolites across different groups, metabolites with similar expression patterns were clustered into distinct subclusters. In each panel, individual lines represent single metabolites, while the blue line indicates the average profile of all metabolites within the subcluster. The red dots on the blue line represent the average scaled abundance values of the corresponding subcluster at each group point. The x-axis represents sample or group names, and the y-axis shows the scaled expression levels of metabolites after data preprocessing and normalization.

In this study, metabolite responses to MeJA concentration were classified into four patterns: subcluster 1 first increased and then decreased, reaching a maximum at 80 mg·L^−1^; subcluster 2 first decreased and then increased, reaching a minimum at 80 mg·L^−1^; subcluster 3 showed a more complex pattern of increase, decrease, and increase again, reaching a maximum at 40 mg·L^−1^ and a minimum at 160 mg·L^−1^; and subcluster 4 increased continuously with increasing MeJA concentration (Figure 8).

#### 2.2.4. Enrichment Analysis of Metabolic Pathways

**Figure 9:**
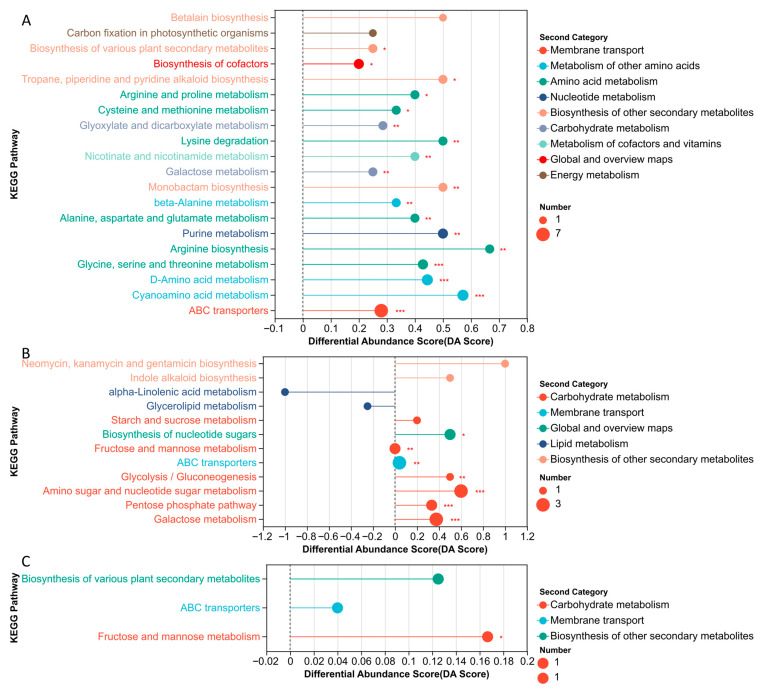
Differential abundance score plots of enriched KEGG pathways. (**A**) Subcluster 1; (**B**) Subcluster 2; (**C**) Subcluster 3. The x-axis represents the differential abundance score (DA score), and the y-axis represents KEGG pathway names. The DA score reflects the overall change trend of all metabolites within a pathway, where a value of 1 indicates that all annotated differential metabolites in the pathway are upregulated, while −1 indicates that all are downregulated. The length of the line segment represents the magnitude of the DA score. The size of each point corresponds to the number of differential metabolites enriched in the pathway. Points distributed on the right side of the zero line indicate an overall upregulation trend, whereas those on the left indicate an overall downregulation trend. Asterisks indicate statistical significance levels: * *p* < 0.05, ** *p* < 0.01, *** *p* < 0.001.

Metabolite sets were established for the subclusters showing distinct response patterns to MeJA concentration. Among them, the first three subclusters contained KEGG annotation information and were therefore selected for KEGG pathway enrichment analysis. The results were visualized using differential abundance (DA) score plots (Figure 9).

A total of 19 pathways were significantly enriched in subcluster 1. These pathways were associated mainly with membrane transport, amino acid metabolism, translation, nucleotide metabolism, and the biosynthesis of other secondary metabolites, together with several pathways related to carbohydrate metabolism and the metabolism of cofactors and vitamins. Representative enriched pathways included ABC transporters, nicotinate and nicotinamide metabolism, cyanoamino acid metabolism, D-amino acid metabolism, arginine biosynthesis, alanine and aspartate and glutamate metabolism, beta-alanine metabolism, aminoacyl-tRNA biosynthesis, and purine metabolism (Figure 9A).

In subcluster 2, seven pathways reached significant enrichment, with the main signals concentrated in carbohydrate metabolism and membrane transport. The carbohydrate-related pathways comprised galactose metabolism, the pentose phosphate pathway, amino sugar and nucleotide sugar metabolism, glycolysis/gluconeogenesis, and fructose and mannose metabolism, whereas ABC transporters represented the major membrane transport pathway in this subcluster (Figure 9B).

The pathways enriched in subcluster 3 were also mainly associated with carbohydrate metabolism, particularly fructose and mannose metabolism (Figure 9C).

## 3. Discussion

The optimized culture system obtained in this study indicates that *V. fauriei* adventitious roots can achieve efficient biomass growth in liquid suspension culture, and that salt concentration, carbon source concentration, and nitrogen concentration had stronger effects than phosphorus concentration. This result is similar to the finding of Sun et al. [30], who reported that medium strength, nitrogen concentration, and sucrose concentration had more significant effects on terpene synthesis in plant roots than phosphorus concentration. Gehlot et al. [17] also showed in liquid-cultured adventitious roots of *Valeriana jatamansi* that medium strength, sucrose, and auxin treatment are key factors affecting biomass accumulation and active constituent accumulation. In addition, the suitable sucrose, total nitrogen, and total phosphorus concentrations obtained here were similar to those reported by Li et al. [18] for *V. officinalis*, which may suggest that congeneric species share partly similar nutrient requirements. At the same time, the culture system obtained here produced a slightly higher proliferation coefficient within a similar culture period. However, the sucrose concentration suitable for *Valeriana jatamansi* in the study of Gehlot et al. [17] was clearly lower than that suitable for *V. fauriei* in the present study, suggesting that different species within the same genus still differ in their sensitivity to culture conditions. This agrees with the view of Yaseen et al. [31] that carbohydrates are crucial for in vitro morphogenesis, which itself is an energy-demanding process, and that carbohydrate requirements vary among culture stages and species. Overall, the *V. fauriei* adventitious root suspension culture system established here follows the general requirements reported for plant adventitious root culture, reflects both similarities and differences in optimal nutrient conditions among congeneric species, and can provide a basis for large-scale biomass accumulation of *V. fauriei* adventitious roots and for further studies on the metabolic effects of MeJA concentration.

These metabolomic results indicate that the response of *V. fauriei* adventitious roots to MeJA was concentration-dependent rather than simply linear. Similar elicitor dose–response patterns have been reported in other plant systems. In the study by Wang et al. [24] on the volatile metabolome of carrot under different MeJA doses, several trend types similar to those observed here were also reported. The degradation of chlorophyll in apple fruit during ripening under different MeJA concentrations also showed a partly similar regulation pattern to subclusters 1 and 2, in which low concentrations promoted but high concentrations inhibited some metabolites, whereas high concentrations promoted but low concentrations inhibited others [32]. These examples are cited only to show that different MeJA concentrations can produce trend patterns similar to those observed here, and do not imply that the same metabolite classes were involved. Specifically, subcluster 1 consisted mainly of amino acids and related primary metabolites and reached its maximum at 80 mg·L^−1^ MeJA. This may be similar to the finding of Hyeon et al. [25], who reported that MeJA treatment can reorganize major metabolites including central carbon compounds, amino acids, and sugars, thereby increasing the levels of some amino acids and sugars at an appropriate concentration. In contrast, subcluster 2 consisted mainly of sugar-related compounds and reached its minimum at 80 mg·L^−1^ MeJA. Sagharyan et al. [33] found that an appropriate MeJA concentration can redirect the dynamics of free sugars and amino acids in *Linum album* cells, and these compounds can serve as energy sources and carbon skeletons for amino acid biosynthesis, thereby promoting lignan accumulation. This suggests that, under 80 mg·L^−1^ MeJA treatment, compounds in subcluster 2 may be more readily consumed as precursors for further synthesis of secondary metabolites.

Previous studies have shown that the binding of asparagine to transfer RNA can activate the aminoacyl-tRNA biosynthesis pathway, and the strong demand for amino acids in this pathway may drive cells to absorb environmental nitrogen sources to synthesize new amino acids [25,34], which is consistent with the simultaneous enrichment of aminoacyl-tRNA biosynthesis and multiple amino acid metabolism pathways in subcluster 1 in the present study. Sucrose is degraded into glucose and fructose, and glucose is used more widely than fructose in plant metabolism [25]. This may explain why carbohydrate metabolism showed relatively high abundances in both subcluster 1 and subcluster 2, even though their overall trends were opposite. Savchenko et al. [35] noted that many plant secondary metabolites originate from aromatic compounds and amino acids, highlighting their linking role between primary and secondary metabolism. Based on this view, it may be inferred that the marked decrease in benzenoid compounds at 80 mg·L^−1^ resulted from their consumption as precursors for the further synthesis of secondary metabolites, which in turn led to significant up-regulation of pathways related to the biosynthesis of other secondary metabolites. Compared with subcluster 2, subcluster 3 was also related to carbohydrate metabolism, but its extreme points were different, suggesting that even within the same metabolic network, different components differ in their sensitivity to MeJA. Although no related pathway was annotated in KEGG for subcluster 4, HMDB and CAS ID annotations showed that all compounds in this cluster were lipids and lipid-like molecules. Welling et al. [36] found a dose-dependent response of cannabinoids to MeJA in *Cannabis sativa*, which is consistent with the increase in some lipids and lipid-like molecules with increasing MeJA concentration in the present study.

In summary, methyl jasmonate exerted a complex and fine regulatory effect on the metabolism of adventitious roots of *V. fauriei*. This effect was reflected not only in the activation or inhibition of metabolic pathways, but also in the redistribution of dynamic balance and metabolic flux within the plant. As the core repressors and coreceptors of JA signaling, JAZ proteins are widely involved in plant growth, stress responses, and metabolic regulation, and serve as important hubs linking plant growth, defense, and metabolic reprogramming [37]. Previous studies showed that in the jasmonic acid signaling pathway, JAZ proteins are degraded through COI1-mediated regulation and at the same time show increased gene expression induced by the transcription factor MYC2, forming a dynamic feedback network with stage-dependent features. As a result, plant responses to JA or MeJA also show dynamic stage-dependent characteristics [38]. The diverse metabolic response patterns observed in this study may therefore reflect the regulation of adventitious root metabolism in *V. fauriei* by the MeJA signaling network mediated by JAZ proteins. However, based on the present metabolomic data, it cannot be directly concluded that the JAZ/MYC2 module was activated in *V. fauriei* adventitious roots, and this mechanism still needs further confirmation by transcriptomic analysis. Even so, the present metabolomic evidence suggests that 80 mg·L^−1^ MeJA was the treatment concentration with the most pronounced effect on metabolic biosynthesis in *V. fauriei* adventitious roots.

## 4. Materials and Methods

### 4.1. Experimental Materials

#### 4.1.1. Plant Materials

The plant material used in this study consisted of in vitro adventitious root segments of *V. fauriei* that had been repeatedly subcultured in the laboratory. The source flowering plants were identified as *V. fauriei* of the genus *Valeriana*, family Valerianaceae, by Professor Xueli Quan, College of Agriculture, Yanbian University.

#### 4.1.2. Chemicals and Reagents

Table 3 lists the major reagents and consumables used in this study, along with their catalog numbers and manufacturers.

### 4.2. Proliferation Culture of Adventitious Roots of Valeriana fauriei Briq.

#### 4.2.1. Screening of Media for Adventitious Root Proliferation

Root tips (2 cm) of *V. fauriei* were excised and inoculated into four prepared media: Woody Plant Medium (WPM), Murashige and Skoog medium (MS), White’s medium (White), and Chu’s N6 medium (N6), all containing 30 g·L^−1^ sucrose and adjusted to pH 5.7. Ten root tips were inoculated into each flask, and each medium had four replicates. The inoculum amount was weighed before inoculation. The culture conditions were 25 °C, 100 r·min^−1^, and dark conditions.

After 28 d, the fresh weight of adventitious roots was measured, and the proliferation coefficient in suspension culture was calculated using the following Equation (2).

Proliferation coefficient = (harvested amount − inoculum amount)/inoculum amount(2)

#### 4.2.2. Uniform Design of Adventitious Root Suspension Culture

A four-factor, ten-level uniform design was used for adventitious root suspension culture according to Table 4. Sucrose concentration, total nitrogen concentration, total phosphorus concentration, and salt solution concentration were selected as the four factors. The optimal medium screened in Section 4.2.1 was used as the basal medium, and its salt solution concentration was set as ×. The variation range of salt solution concentration was 0.5×–2.75×, that of total nitrogen concentration was 4.8–94.8 mmol·L^−1^, that of total phosphorus concentration was 0.75–3 mmol·L^−1^, and that of sucrose concentration was 25–52 g·L^−1^. The specific levels and experimental scheme are shown in Table 5.

The optimal medium screened from the medium screening experiment was used as the basal medium. According to Table 4, the sucrose concentration, total nitrogen concentration, total phosphorus concentration, and salt solution concentration were adjusted, whereas the other components remained unchanged. In addition, because adventitious roots after repeated subculture grew more slowly than newly induced roots, 0.2 mg·L^−1^ NAA was added to the medium. The root inoculation method and culture conditions were the same as those in Section 4.2.1. After 28 d of culture, the proliferation coefficient was calculated using Equation (1) and analyzed.

### 4.3. Effects of MeJA on Metabolites in Suspension-Cultured Adventitious Roots of Valeriana fauriei Briq.

#### 4.3.1. MeJA Treatment

The MeJA treatment groups were K0, K1, K2, K3, and K4 at 0, 40, 80, 160, and 320 mg·L^−1^, respectively, with three replicates for each treatment. Samples were harvested after 7 d, rapidly frozen in liquid nitrogen for 15 min, and stored on dry ice for later use.

#### 4.3.2. Preparation of the n-Alkane Standard Solution

A total of 770 μL n-hexane was placed into a 1.5 mL centrifuge tube. Appropriate amounts of the commercial mixed standard C_10_–C_25_ and the n-alkane standards C_26_, C_27_, C_28_, C_29_, C_30_, C_31_, C_32_, and C_33_ were added in sequence and mixed by vortexing to obtain a mixed stock solution of C_10_–C_33_ n-alkanes (50 μg·mL^−1^). The stock solution was diluted to 10 μg·mL^−1^ and analyzed together with the samples.

#### 4.3.3. Sample Pretreatment

Sample pretreatment was carried out by Shanghai Majorbio Bio-pharm Technology Co., Ltd. (Majorbio, Shanghai, China) following its standard workflow for untargeted GC–MS metabolomics. Briefly, 50 mg of each sample was transferred into a centrifuge tube, and 0.5 mL of methanol/water (CH_3_OH:H_2_O = 4:1, *v*/*v*) containing 0.02 mg·mL^−1^ ribitol as an internal standard was added. After one steel bead was introduced, the sample was homogenized at −20 °C for 3 min at 50 Hz (Wonbio-96c multi-sample cryogenic grinder, Shanghai Wanbai Biotechnology Co., Ltd., Shanghai, China). Subsequently, 200 μL chloroform was added, and grinding was continued for another 3 min. The homogenate was subjected to ultrasonic extraction for 30 min and then kept at −20 °C for 30 min (SBL-10DT ultrasonic cleaner, Ningbo Xinzhi Biotechnology Co., Ltd., Ningbo, China). After centrifugation at 13,000 rcf and 4 °C for 15 min (Centrifuge 5424 R high-speed refrigerated centrifuge, Eppendorf, Hamburg, Germany), the supernatant was transferred to a glass derivatization vial and dried under nitrogen (JXDC-20 nitrogen evaporator, Shanghai Jingxin Industrial Development Co., Ltd., Shanghai, China). Then, 80 μL of methoxyamine hydrochloride in pyridine solution (15 mg·mL^−1^) was added, and the mixture was vortexed for 2 min, followed by oximation at 37 °C for 90 min in a shaking incubator. Afterward, 80 μL of BSTFA derivatization reagent containing 1% TMCS was added. The sample was vortexed for 2 min, reacted at 70 °C for 60 min, and then left at room temperature for 30 min prior to GC-MS metabolomic analysis.

#### 4.3.4. GC-MS Analysis

GC-MS analysis was performed using an Orbitrap Exploris GC gas chromatography-mass spectrometry system (Thermo Fisher Scientific, Bremen, Germany).

Derivatized samples were analyzed in split injection mode with an injection volume of 1 μL and a split ratio of 10:1. Chromatographic separation was achieved on a TG-5SILMS capillary column (30 m × 0.25 mm × 0.25 μm; 26096-1420; Thermo Fisher Scientific, Waltham, MA, USA), after which the analytes entered the mass spectrometer. The injector temperature was maintained at 300 °C. High-purity helium was used as the carrier gas at a flow rate of 1.0 mL·min^−1^, and the purge flow was set at 3 mL·min^−1^. The oven temperature program started at 80 °C with no holding time, then increased to 310 °C at a rate of 20 °C·min^−1^ and was held for 8 min. The total run time was 20 min, and the solvent delay was set to 2 min.

Mass spectrometric conditions are shown in Table 6.

#### 4.3.5. Quality Control

To ensure the stability of the analytical system during instrumental analysis, three quality control (QC) samples were prepared in this study. The QC samples were obtained by mixing all samples to be tested, and the subsequent processing procedure was exactly the same as that used for the formal samples. During instrumental analysis, these three QC samples were inserted at intervals.

### 4.4. Data Analysis

#### 4.4.1. Data Analysis for Adventitious Root Proliferation Culture

For the medium screening experiment of adventitious root proliferation, SPSS 26 was used to calculate standard deviations and perform analysis of variance. In the uniform design experiment for adventitious root proliferation culture, sucrose concentration (g·L^−1^), total nitrogen concentration (mmol·L^−1^), total phosphorus concentration (mmol·L^−1^), and salt solution concentration (the original formulation was set as *x*) were defined as *x*_1_, *x*_2_, *x*_3_, and *x*_4_, respectively. Python 3.13 was used for data preprocessing and analysis, and the optimal value was obtained by combining the fitted regression model with the gradient descent method.

#### 4.4.2. Metabolomic Analysis of the Effects of MeJA on Adventitious Roots of *Valeriana fauriei* Briq.

The raw GC-MS files were processed for library identification and data preprocessing using Thermo Compound Discovery 3.3 SP3. The main databases used for library searching were NIST-2023, GC-Orbitrap Metabolomics Library_v2, and the self-built database of Shanghai Majorbio Bio-pharm Technology Co., Ltd. A three-dimensional data matrix in xlsx format was exported, including sample name, metabolite name, peak area, and other information. The matrix was uploaded to the Majorbio Cloud Platform (https://cloud.majorbio.com, accessed on 15 April 2026) for missing-value simulation, data normalization, log transformation, and subsequent analyses.

## 5. Conclusions

The suitable conditions for suspension culture of adventitious roots of *V. fauriei* were as follows: WPM as the basal medium, 42 g·L^−1^ sucrose, 44.8 mmol·L^−1^ total nitrogen, 1.5 mmol·L^−1^ total phosphorus, 1.8× salt solution concentration relative to the original medium, 0.2 mg·L^−1^ NAA, and pH 5.7. Under dark conditions at 25 °C and 100 r·min^−1^ for 28 d, the proliferation coefficient reached 27.07. GC-MS-based metabolomics further identified 379 metabolites across the five MeJA treatment groups, including 181 significant differential metabolites. Among them, 80 mg·L^−1^ MeJA most strongly affected pathways related to membrane transport, amino acid metabolism, translation, nucleotide metabolism, and the biosynthesis of other secondary metabolites. These findings provide a useful in vitro platform for increasing biomass and metabolite yield in *V. fauriei* adventitious roots, and also offer a theoretical basis for further understanding jasmonate signaling in the regulation of adventitious root metabolism in *Valeriana* species.

## Figures and Tables

**Table 1:** Screening of Media for Adventitious Root Proliferation of *Valeriana fauriei* Briq.

Medium	Proliferation Coefficient
WPM	25.01 ± 4.48 a
MS	0.88 ± 0.54 d
White	6.51 ± 1.74 c
N6	17.78 ± 3.87 b

WPM, Woody Plant Medium; MS, Murashige and Skoog medium; White, White medium; N6, Chu’s N6 medium. Values are presented as mean ± SD. Different lowercase letters indicate significant differences among media at *p* < 0.05.

**Table 2:** *t*-Test Results of the Uniform Design for Adventitious Root Suspension Culture.

Variable	Regression Coefficient	Standard Error	t Value	*p* Value	Significance
*x*_1_	0.25	0.05	5.00	0.002	**
*x*_2_	0.15	0.04	3.75	0.010	**
*x*_3_	−2.50	1.10	−2.27	0.085	
*x*_4_	1.80	0.50	3.60	0.012	*

* *p* < 0.05; ** *p* < 0.01.

**Table 3:** Major Reagents Used in This Study.

Reagents and Consumables	Catalog No.	Manufacturer
n-Pentadecane-d32	D874357-100 mg	Macklin, Shanghai, China
n-Alkane Standard Mixture (C10–C25)	1069335HE	LGC, Teddington, UK
Hexacosane	BZRXD-KE	TCI, Tokyo, Japan
Heptacosane	DJQOK-KI	TCI, Tokyo, Japan
Octacosane	74684-250MG	Sigma-Aldrich, St. Louis, MO, USA
Nonacosane	BCCF8352	Sigma-Aldrich, St. Louis, MO, USA
n-Triacontane	MKCJ4572	Sigma-Aldrich, St. Louis, MO, USA
n-Hentriacontane	G1050290	LGC, Teddington, UK
n-Dotriacontane	BCBW0661	Sigma-Aldrich, St. Louis, MO, USA
n-Tritriacontane	BCCFC6180	Sigma-Aldrich, St. Louis, MO, USA
Methyl jasmonate (MeJA)	C16939292	Macklin, Shanghai, China

**Table 4:** U10(10^8^) Design Table.

s	Column No.						D
2	1	6					0.1125
3	1	5	6				0.1681
4	1	3	4	5			0.2236
5	1	3	4	5	7		0.2414
6	1	2	3	5	6	8	0.2994

**Table 5:** Uniform Design Scheme for Adventitious Root Suspension Culture of *Valeriana fauriei* Briq.

No.	Sucrose (g·L^−1^)	Total N (mmol·L^−1^)	Total P (mmol·L^−1^)	Salt Solution Concentration
1	25	24.8	1.5	1.5×
2	28	54.8	2.5	2.75×
3	31	84.8	0.75	1.25×
4	34	4.8	1.75	2.5×
5	37	34.8	2.75	1×
6	40	64.8	1	2.25×
7	43	94.8	2	0.75×
8	46	14.8	3	2×
9	49	44.8	1.25	0.5×
10	52	74.8	2.25	1.75×

**Table 6:** Mass Spectrometric Parameters.

Description	Parameter
Scan mode	Full Scan
Scan range	35–500
Resolution	30,000
AGC Target	AGC Target Standard
Maximum injection time	Auto
Ion source temperature (°C)	250
Ion source type	EI
Repeller (V)	10
Default ion source voltage (V)	5
Lens 1 (V)	−50
Lens 2 (V)	−0.5
Lens 3 (V)	−35
Electron lens (V)	15
Electron energy (eV)	70
Emission current (μA)	50

## Data Availability

The original GC-MS raw data for all samples generated in this study have been deposited in Figshare. A DOI has been reserved for the dataset: [10.6084/m9.figshare.32063478]. During peer review, the data are available to the editors and reviewers via a private review link. The dataset will be made publicly available upon publication.
